# Author Correction: Control over the fibrillization yield by varying the oligomeric nucleation propensities of self-assembling peptides

**DOI:** 10.1038/s42004-020-00436-4

**Published:** 2020-11-27

**Authors:** Chun Yin Jerry Lau, Federico Fontana, Laurens D. B. Mandemaker, Dennie Wezendonk, Benjamin Vermeer, Alexandre M. J. J. Bonvin, Renko de Vries, Heyang Zhang, Katrien Remaut, Joep van den Dikkenberg, João Medeiros-Silva, Alia Hassan, Barbara Perrone, Rainer Kuemmerle, Fabrizio Gelain, Wim E. Hennink, Markus Weingarth, Enrico Mastrobattista

**Affiliations:** 1 Utrecht Institute for Pharmaceutical Sciences, Department of Pharmaceutics, Faculty of Science, Utrecht University, Universiteitsweg 99, 3584 CG Utrecht, The Netherlands; 2 IRCCS Casa Sollievo della Sofferenza, Opera di San Pio da Pietralcina, Viale Capuccini 1, 71013 San Giovanni Rotondo, Italy; 3 ASST Grande Ospedale Metropolitano Niguarda, Center for Nanomedicine and Tissue Engineering, Piazza dell’Ospedale Maggiore 3, 20162 Milan, Italy; 4 Inorganic Chemistry and Catalysis Group, Debye Institute for Nanomaterials Science, Department of Chemistry, Faculty of Science, Utrecht University, Universiteitsweg 99, 3584 CG Utrecht, The Netherlands; 5 NMR Spectroscopy, Bijvoet Centre for Biomolecular Research, Department of Chemistry, Faculty of Science, Utrecht University, Padualaan 8, 3584 CH Utrecht, The Netherlands; 6 Laboratory of Physical Chemistry and Colloid Science, Wageningen University, Dreijenplein 6, 6703 HB Wageningen, The Netherlands; 7 Ghent Research Group on Nanomedicines, Laboratory of General Biochemistry and Physical Pharmacy, Ghent University, Ottergemsesteenweg 460, 9000 Ghent, Belgium; 8 Bruker BioSpin AG, Industriestrasse 26, 8117 Fällanden, Switzerland

**Keywords:** Peptides, Solid-state NMR, Self-assembly, Bioinspired materials

Correction to: *Communications Chemistry* 10.1038/s42004-020-00417-7, published online 11 November 2020.

The original version of this Article contained an error in Fig. 4, whereby the magenta 2D ^13^C-^13^C spectrum (700 ms mixing time), which relates to the blue assignments, was omitted.

In addition, co-author Federico Fontana was incorrectly affiliated with ASST Grande Ospedale Metropolitano Niguarda.

These errors have now been corrected in both the PDF and HTML versions of the Article.

