# Supplementary material for: Control over the fibrillization yield by varying the oligomeric nucleation propensities of self-assembling peptides
Source: Commun Chem. 2020 Nov 11;3:164. doi: 10.1038/s42004-020-00417-7 (PMC9814929; doi:10.1038/s42004-020-00417-7)
Supplement: Supplementary file 1 — Supplementary Information [file 42004_2020_417_MOESM1_ESM.pdf]

Control over the fibrillization yield by varying the oligomeric nucleation propensities of self-assembling peptides

Chun Yin Jerry Lau, Federico Fontana, Laurens D.B. Mandemaker, Dennie Wezendonk, Benjamin Vermeer, Alexandre M.J.J. Bonvin, Renko de Vries, Heyang Zhang, Katrien Remaut, Joep van den Dikkenberg, João Medeiros-Silva, Alia Hassan, Barbara Perrone, Rainer Kuemmerle, Fabrizio Gelain, Wim E. Hennink, Markus Weingarth* and Enrico Mastrobattista*

Table S1: TANGO and WALTZ scoring

To predict the aggregation and fibrillization propensity of the designer SLPs, the statistical thermodynamics algorithm TANGO and the position scoring matrices WALTZ were used to calculate the respective scores (available at http://tango.crg.es/tango.jsp and http://waltz.switchlab.org/).

A) SLP1: Ac-VVVTILLEE-COOH

**Sequence**	**Ac**	**V**	**V**	**V**	**T**	**I**	**L**	**L**	**E**	**E**
**TANGO**	0.00	96.23	98.61	98.89	98.69	98.69	74.31	27.73	0.00	0.00
**WALTZ**	/	97.66	97.66	97.66	97.66	97.66	97.66	97.66	/	/

B) SLP3: Ac-VVVTLLLEE-COOH

Sequence	Ac	V	V	V	T	L	L	L	E	E
TANGO	0.00	94.98	97,33	97.41	97.41	97.41	73.34	27.36	0.00	0.00
WALTZ	/	86.29	86.29	86.29	86.29	86.29	86.29	86.29	/	/

C) SLP2: Ac-VVVTILLEEE-COOH

Sequence	Ac	V	V	V	T	I	L	L	E	E	E
TANGO	0.00	96.34	99.03	99.17	99.17	99.17	83.80	54.44	0.00	0.00	0.00
WALTZ	/	97.66	97.66	97.66	97.66	97.66	97.66	97.66	/	/	/

D) **SLP4: Ac-VVVTLLLEEE-COOH**

Sequence	Ac	V	V	V	T	L	L	L	E	E	E
TANGO	0.00	95.55	98.21	98.36	98.36	98.36	83.11	53.99	0.00	0.00	0.00
WALTZ	/	86.29	86.29	86.29	86.29	86.29	86.29	86.29	/	/	/

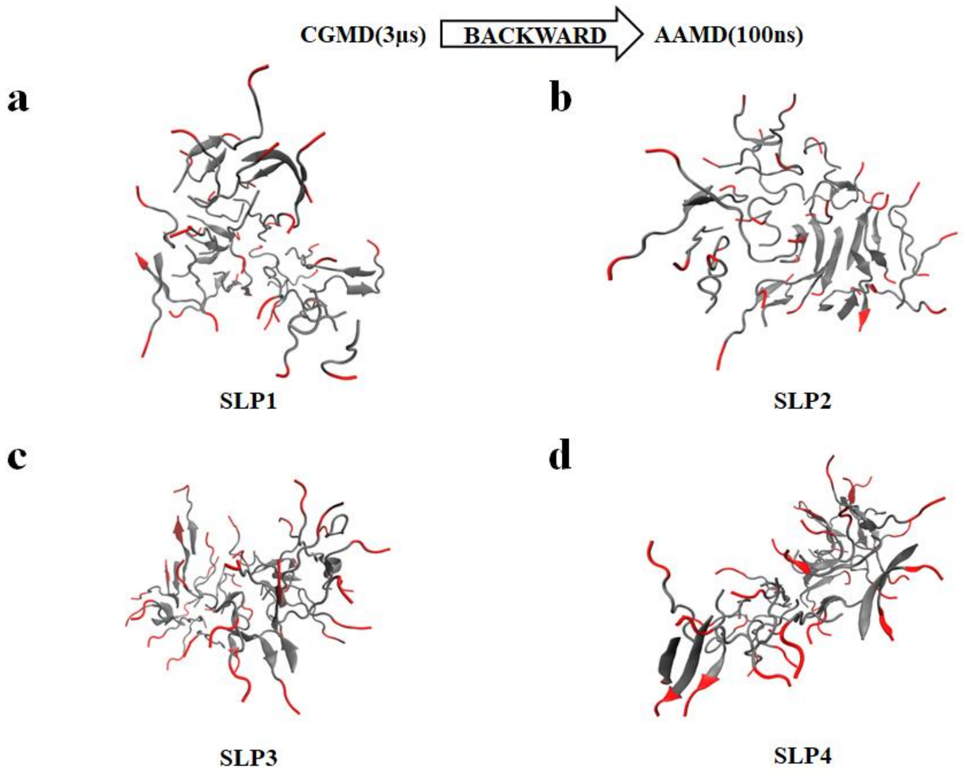

Figure S1. Fine-grained micellar oligomers MD models. Final trajectory of 100ns AAMD simulation after back transformation[1] from CGMD simulation.

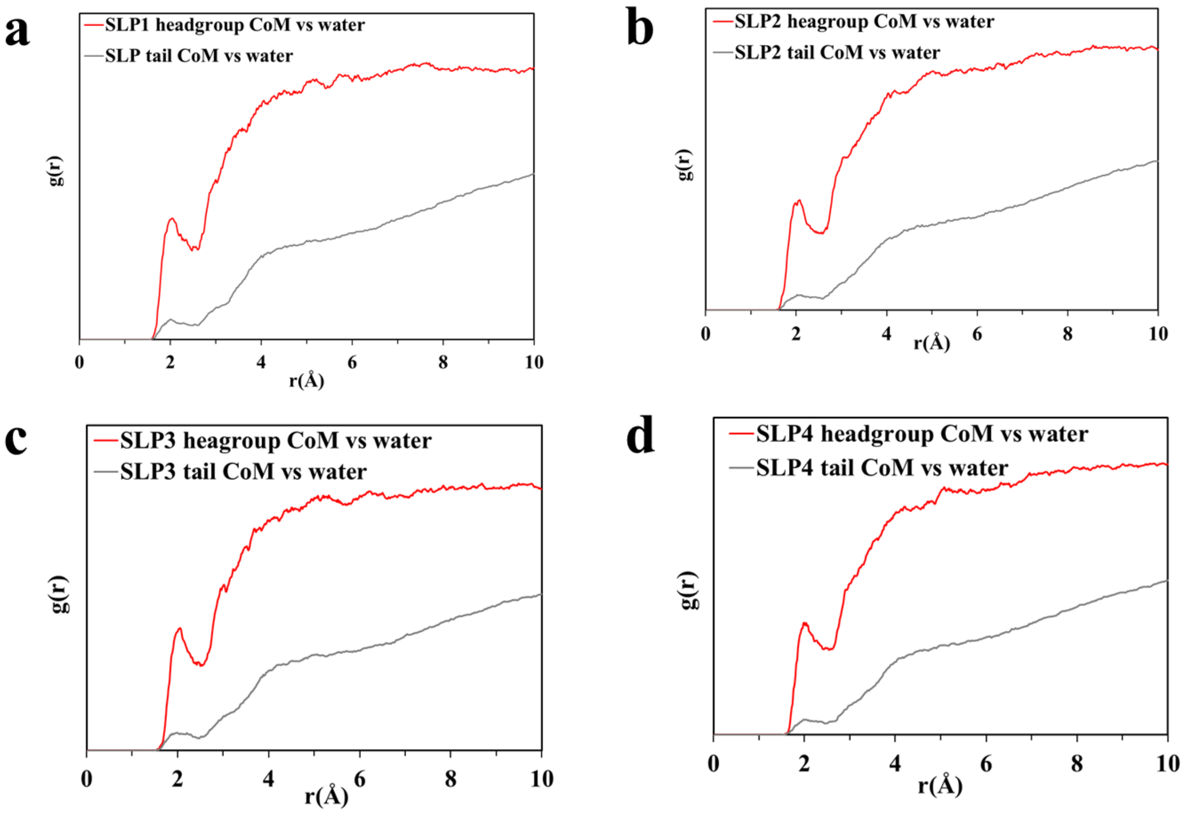

Figure S2. Radial Distribution function (RDF) between Center of Mass (CoM) of tail/headgroup of SLP oligomers and water. RDF functions of CoM of tail/headgroup and water is plotted for the final trajectory of fine-grained MD structures reported in Figure S1. It confirms the micellar arrangement in oligomers---tail clusters were desolvated, headgroups remained solvated.

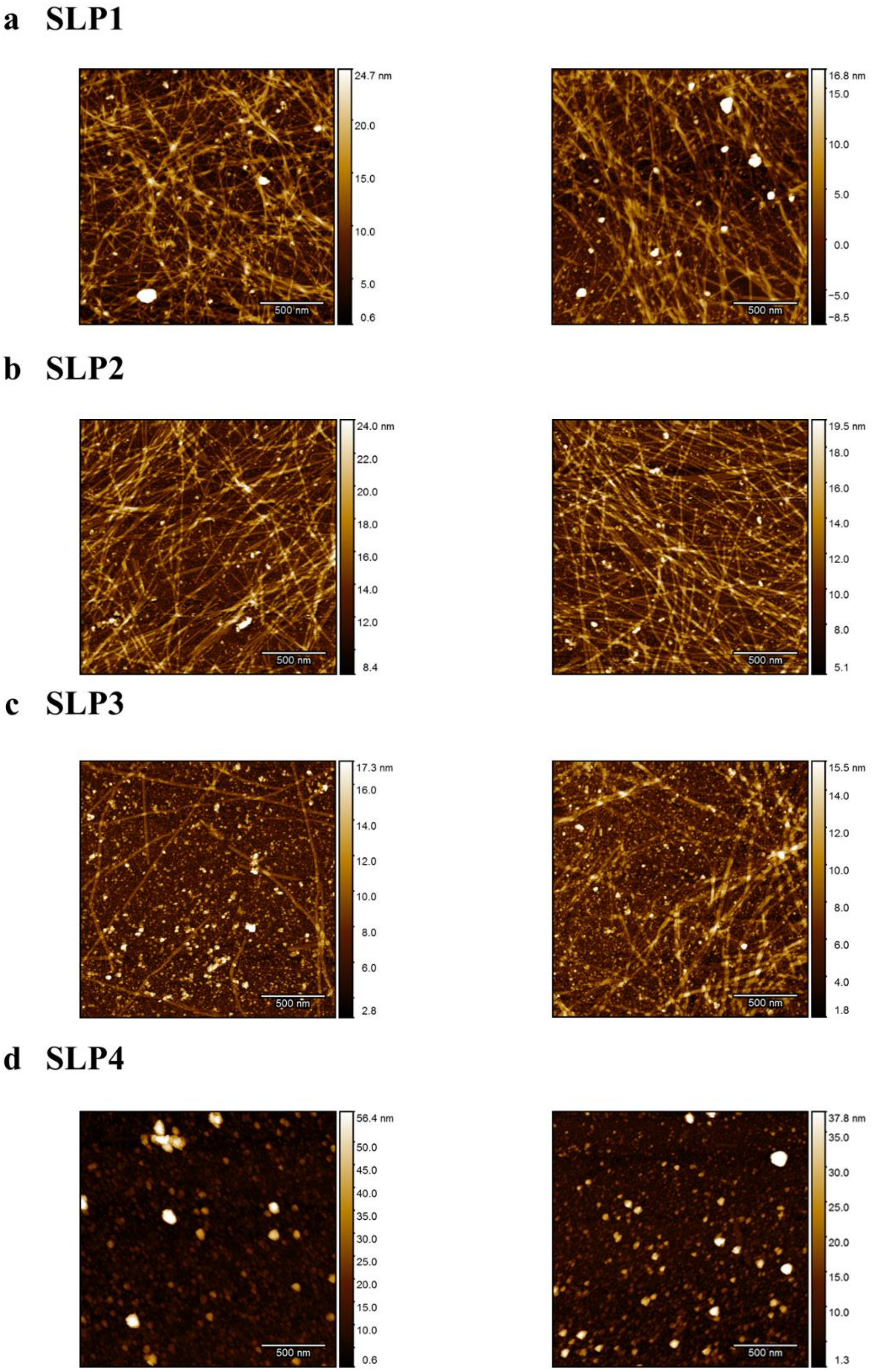

Figure S3. Atomic force microscopy (AFM) imaging. Freshly cleaved mica wafer was incubated for 5 min with 5 times diluted poly-L-lysine solution (150-300 kDa, 0.1%, Sigma-Aldrich, St. Louis, MO) and subsequently washed three times with MQ water. 4mM of peptide solution were diluted tenfold with PBS and a drop (50 µL) of the solution was deposited on the coated mica and incubated for 5 minutes. The mica was washed three times with 500 µL of dH2O to remove salts and dried under a stream of nitrogen. AFM micrographs were recorded using a Bruker MultiMode 8 (ScanAsyst Air silicon nitride probes, spring constant 0.4 N/m, nominal tip radius 2 nm) and post-processed by a plane subtraction and line alignment. Three different spots were measured on the sample to confirm uniformity and get a comprehensive view of the sample's features.

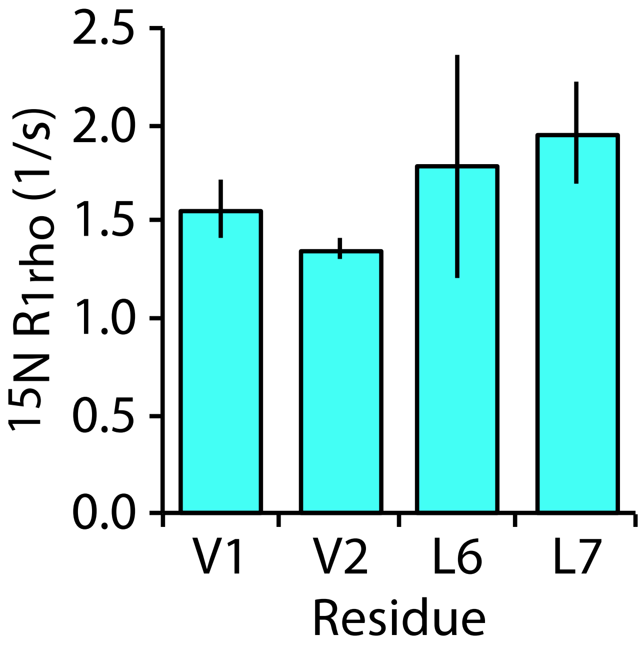

Figure S4. Site-resolved ^{15}N $R1_{rho}$ ssNMR dynamics of the SLP1 fibril. Site-resolved ^{15}N $R1_{rho}$ ssNMR dynamics of assembled SLP1 acquired at 60 kHz MAS and 950 MHz magnetic field (22.3 T ^{1}H frequency). While the very slow relaxation shows that the β-structured residues are tightly assembled, the enhanced dynamics of Leu7 is in line with an interdigitated alignment in which the anionic C-terminus protrudes from the assembly. Spectra were measured with 2D $^{13}C(^{15}N)^{1}H$ experiments. The detour via the ^{13}C-dimension was necessary due to spectral overlap of NH correlations.

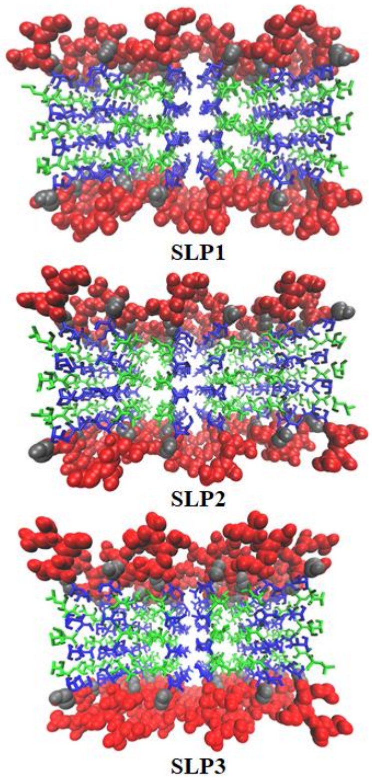

Interface amino acids	ΔGwoct per cross-section (kcal/mol)
Leu6 Thr4 Val2 / Val2 Thr4 Leu6	-2.922
Val1 Val3 Ile5 Leu7 / Leu7 Ile5 Val3 Val1	-6.584
Leu6 Thr4 Val2 / Val2 Thr4 Leu6	-2.922
Val1 Val3 Leu5 Leu7 / Leu7 Leu5 Val3 Val1	-6.84
Leu6 Thr4 Val2 / Val2 Thr4 Leu6	-2.922
Val1 Val3 Ile5 Leu7 / Leu7 Ile5 Val3 Val1	-6.584

Figure S5 Side chain interface assignment. Molecular representation of two chemically anisotropic inter-side chain interfaces assigned for SLP1-3. Averaged hydrophobicity per amino acid and hydrophobicity per single cross-sectional face were calculated as the averaged value of free energies (ΔG, kcal/mol) for transferring specific amino acid from water to n-octanol (woct)[2].

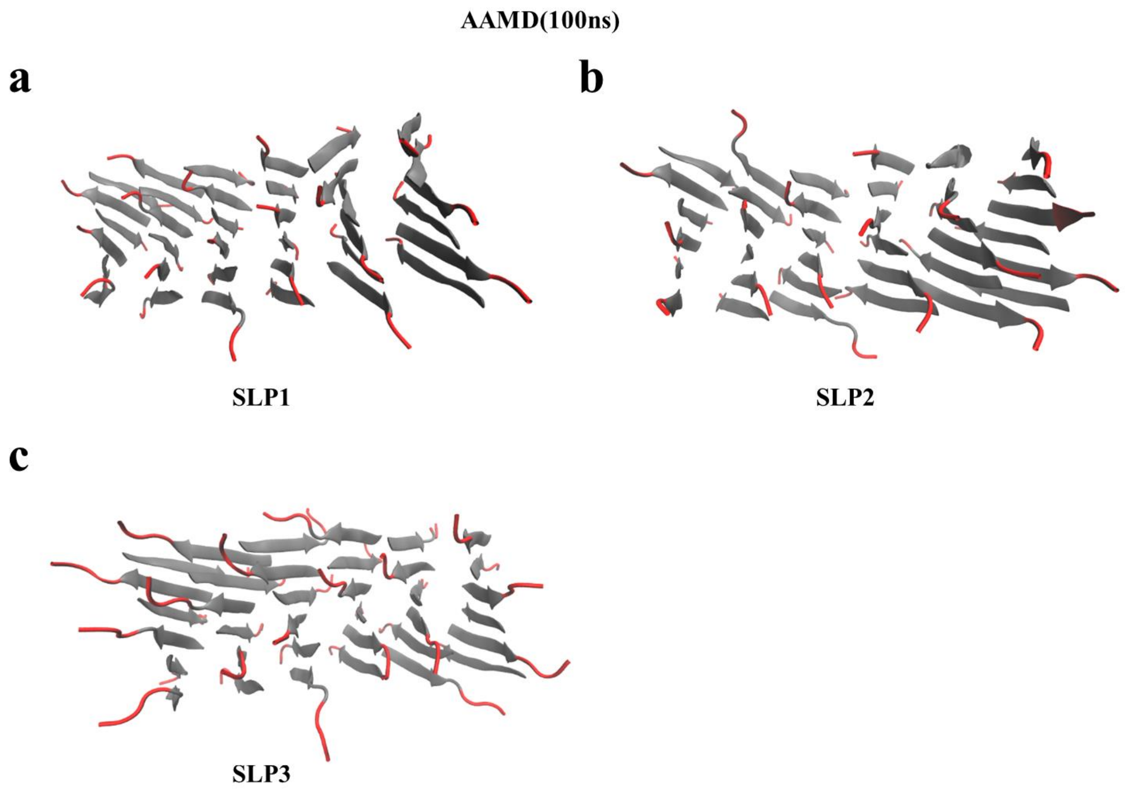

Figure S6 Final (100ns) configuration of the equilibrated fibril molecular models. The chirality of the L-amino acids leads to left-handed twist observed in the SLP models.

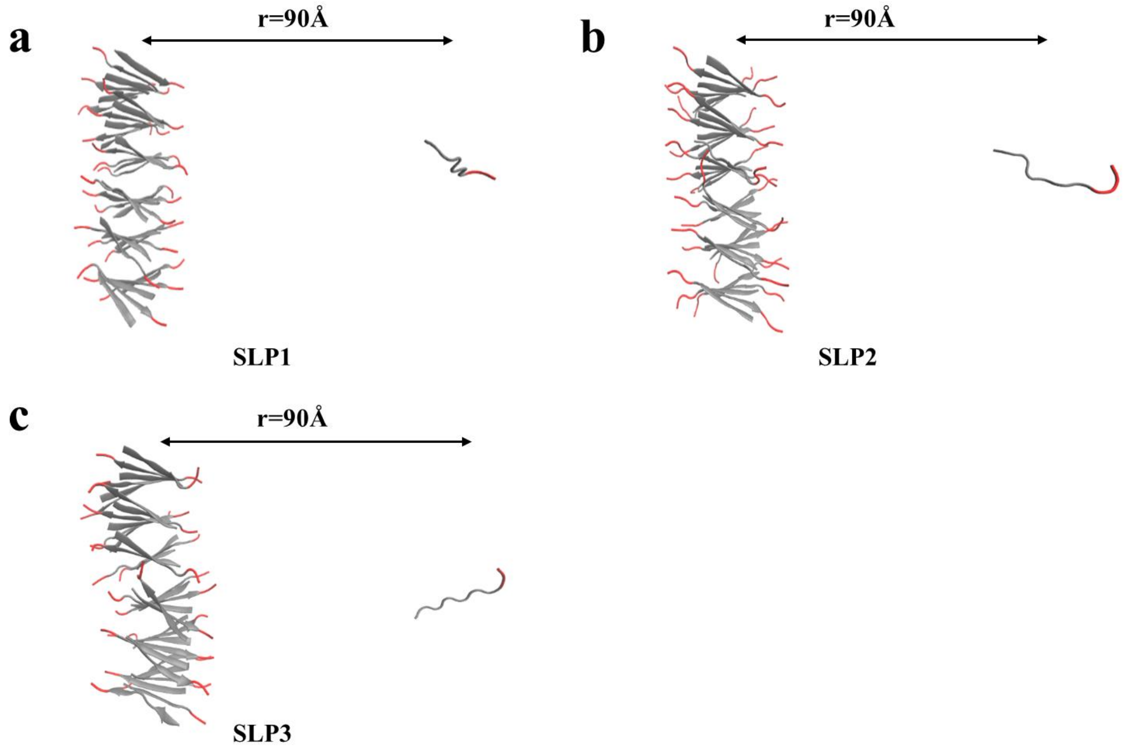

Figure S7 Final trajectory snapshot of Steered MD simulation for SLP fibril structures. One β-sheet SLP was dragged from the core of the fibril models along the reaction coordinate r for 90Å.

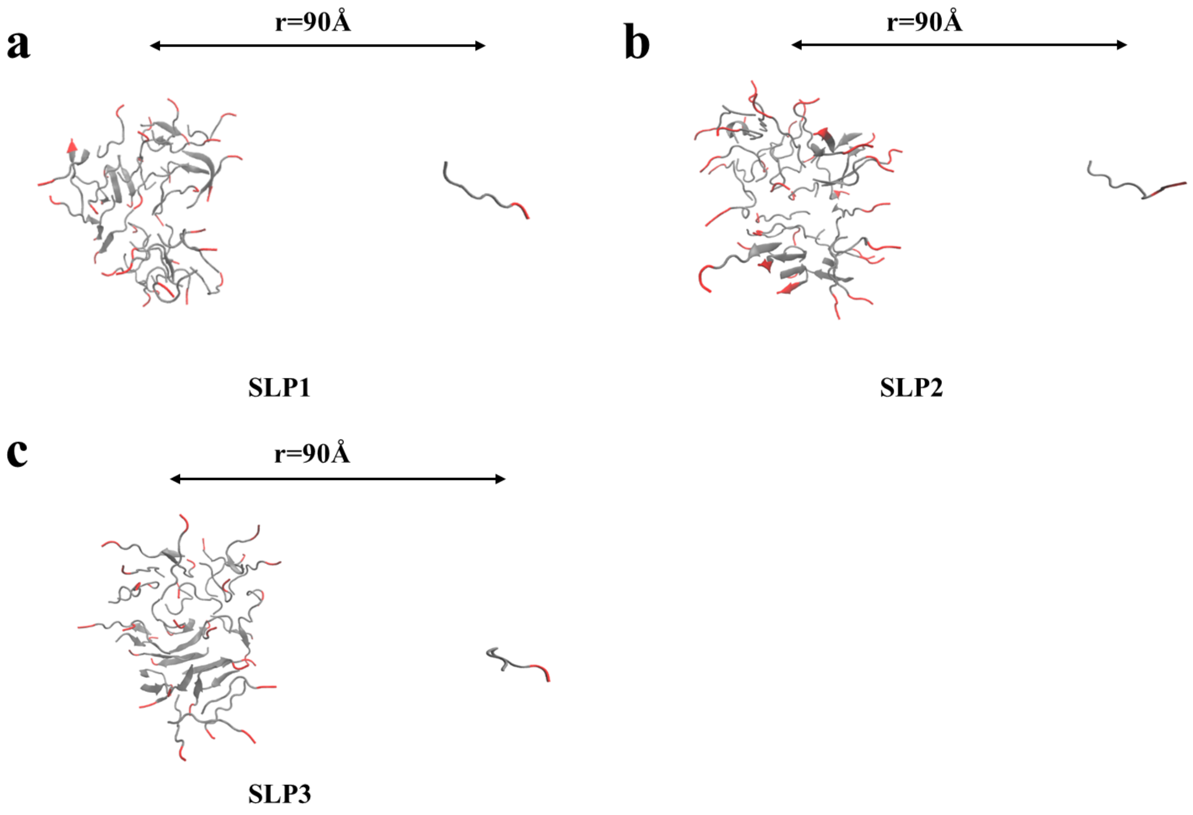

Figure S8 Final trajectory snapshot of Steered MD simulation for SLP oligomeric structures. One random-coiled SLP was dragged from the core of the oligomer models along the reaction coordinate r for 90Å.

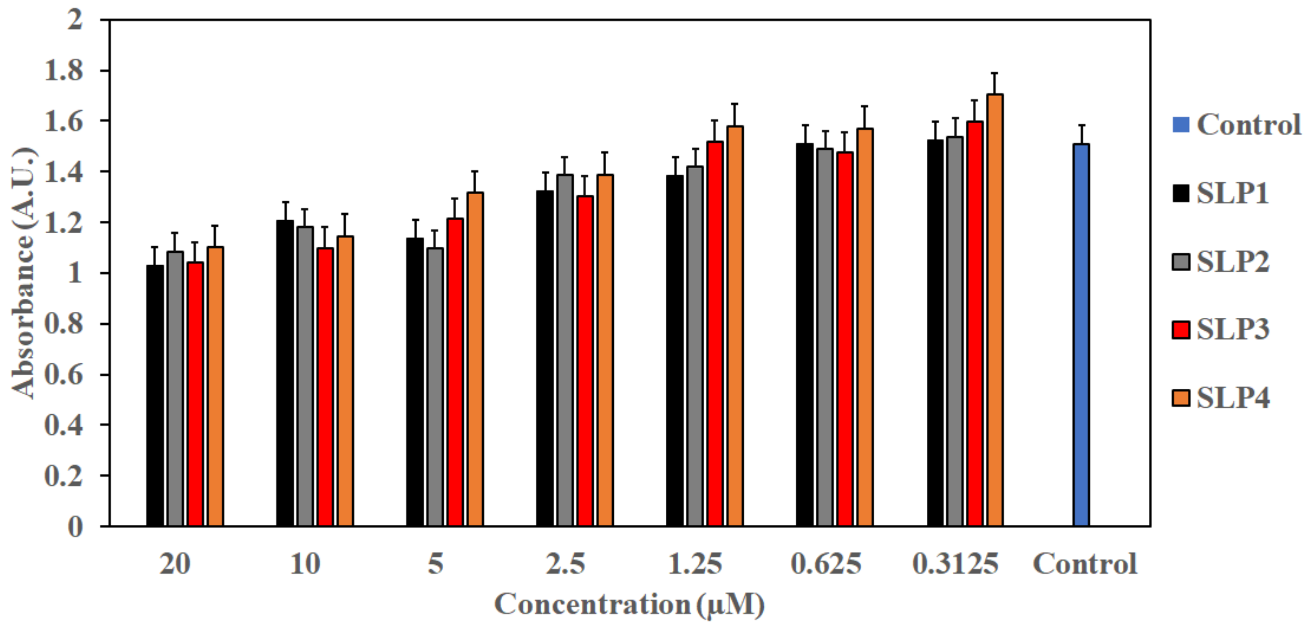

Figure S9 Cell viability of exposing SLP assemblies at biological relevant concentration. A431 cells (5000 cells/well) were exposed to SLP assemblies at a range of concentration (0.3125-20 μM) or phosphate buffered saline (1X, control) for 24 hours. The cell viability was then assessed with an MTS assay (absorbance at 490nm after 2 hours incubation). Results indicates that the working concentration for SLP 1-4 assemblies are recommended to be ≤0.625μM. Values representative as average of a triplicate (mean ± SEM).

Supplementary Note 1-12: HPLC-MS traces of synthesized SLPs

Note S1. SLP1: Ac-VVVTILLEE-COOH

Analytical RP-HPLC

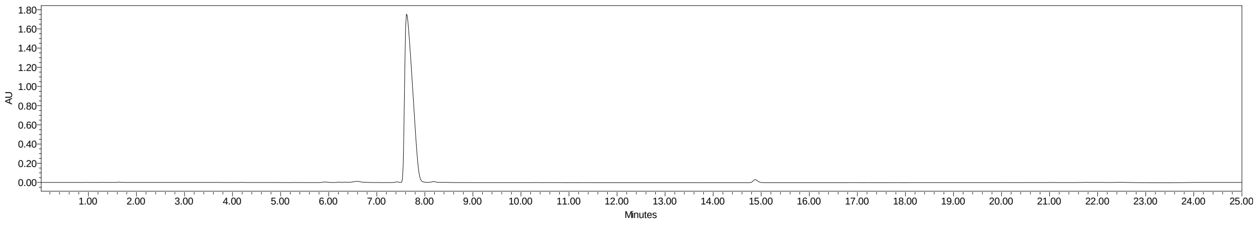

MS

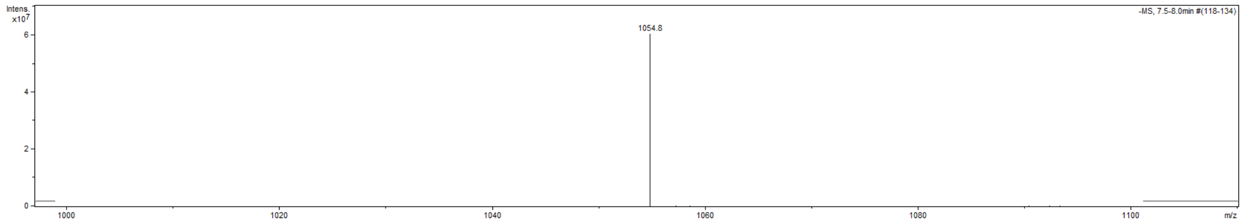

Note S2. SLP2: Ac-VVVTLLLEE-COOH

Analytical RP-HPLC

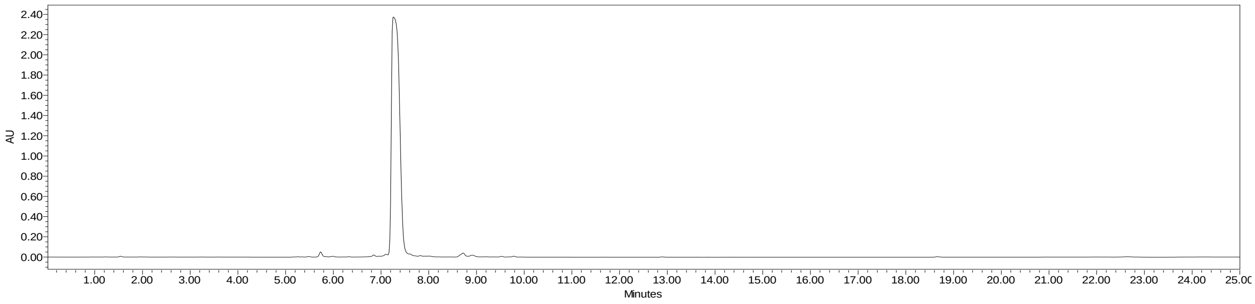

MS

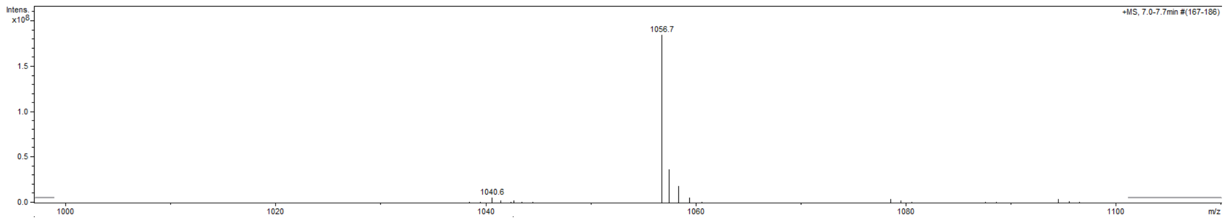

Note S3. SLP3: Ac-VVVTILLEEE-COOH

Analytical RP-HPLC

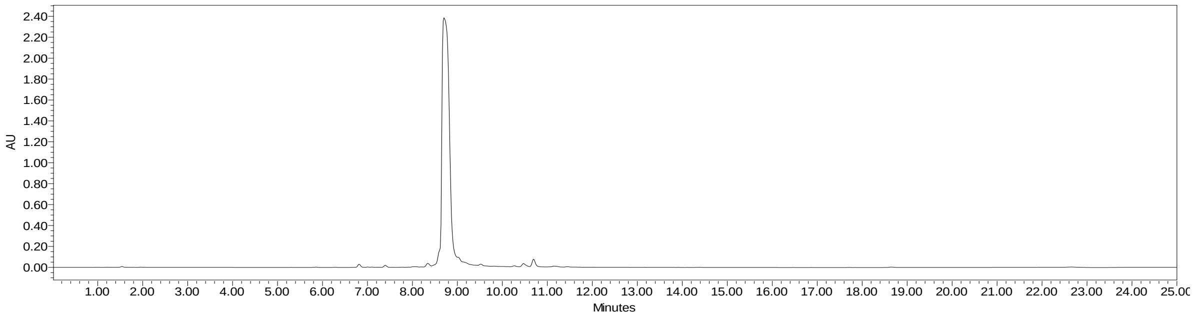

MS

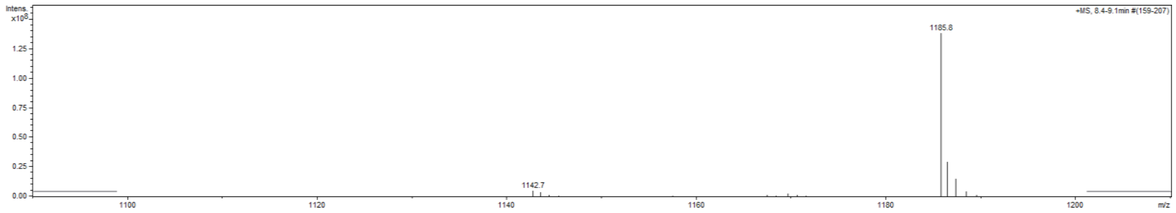

Note S4. SLP4: Ac-VVVTLLLEEE-COOH

Analytical RP-HPLC

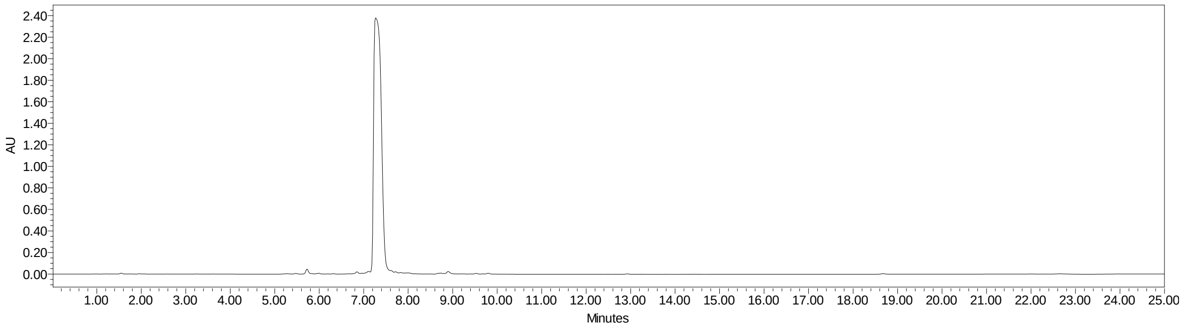

MS

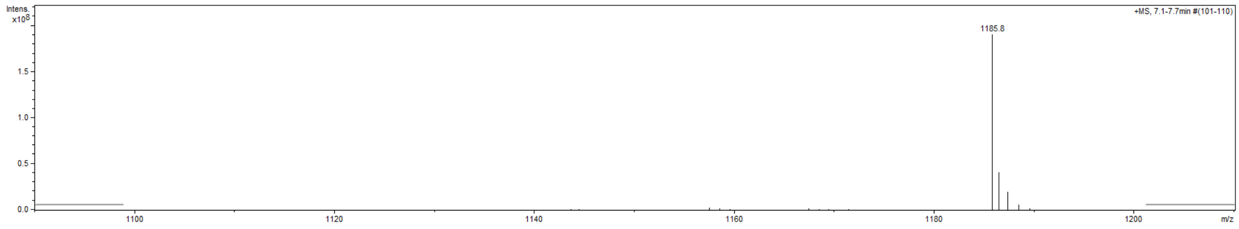

Note S5. SLP1: Ac-VVVTILLEE-COOH (isotopic 13C, 15N labeling)

Analytical RP-HPLC

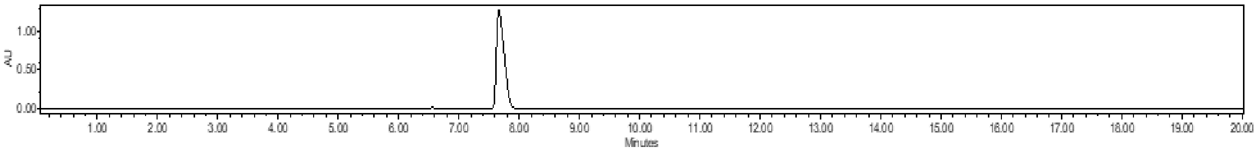

MS

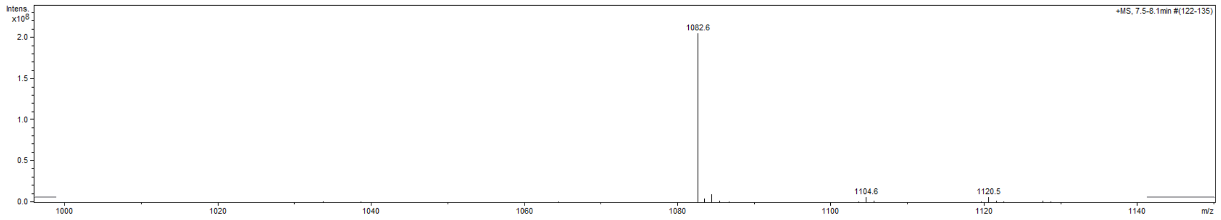

Note S6. SLP2: Ac-VVVTLLLEE-COOH (isotopic 13C, 15N labeling)

Analytical RP-HPLC

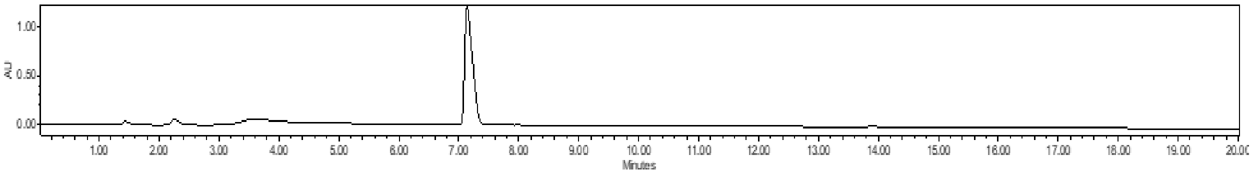

MS

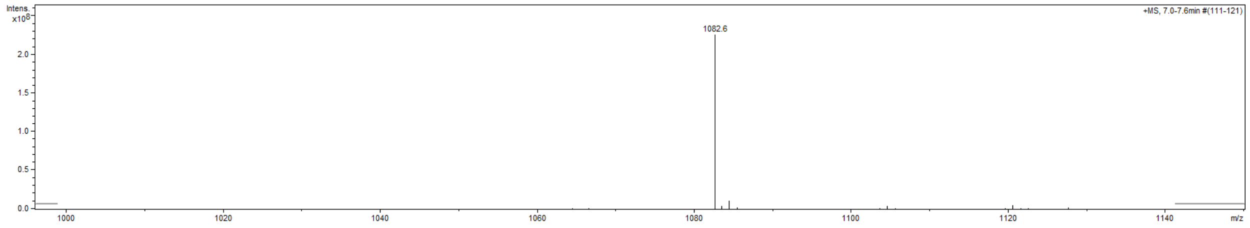

Note S7. SLP3: Ac-VVVTILLEEE-COOH (isotopic 13C, 15N labeling)

Analytical RP-HPLC

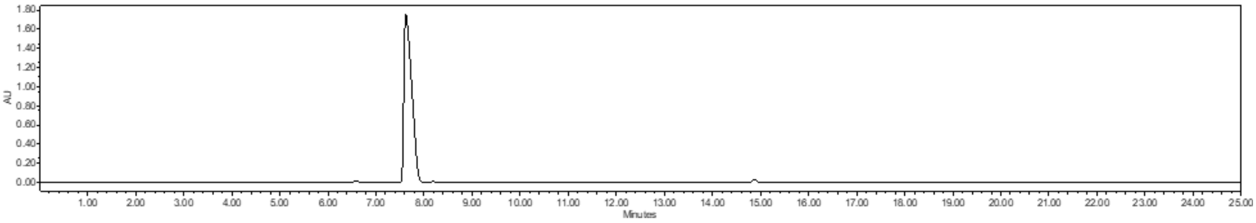

MS

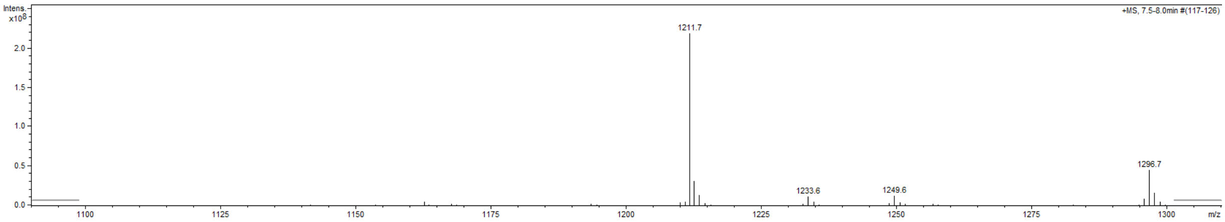

Note S8. SLP4: Ac-VVVTLLLEEE-COOH (isotopic 13C, 15N labeling)

Analytical RP-HPLC

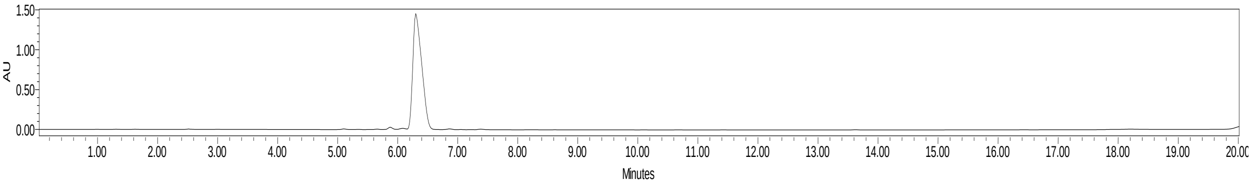

MS

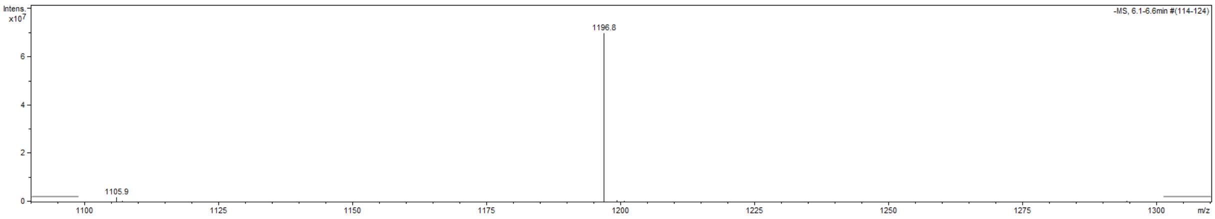

Note S9. SLP1: Ac-VVVTILLEEK(Cy5)-COOH

MS

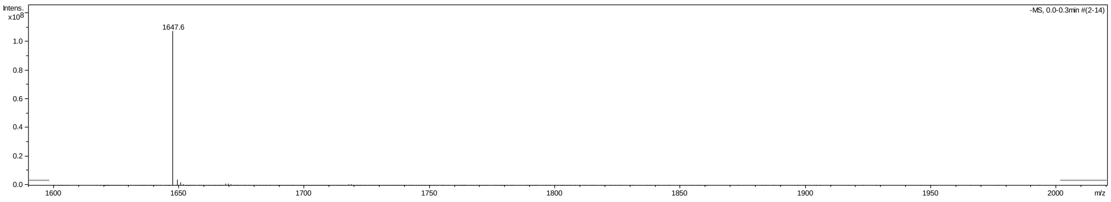

Note S10. SLP2: Ac-VVVTLLLEEK(Cy5)-COOH

MS

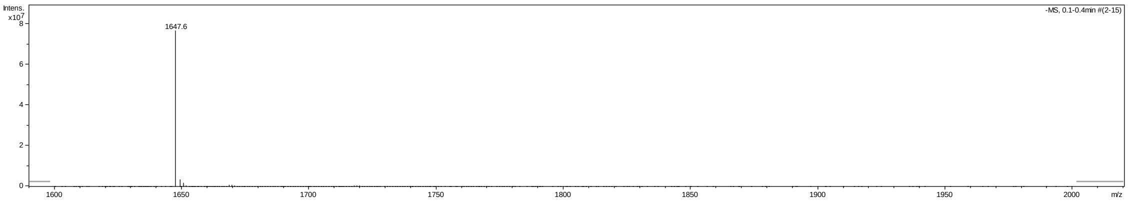

Note S11. SLP3: Ac-VVVTILLEEEK(Cy5)-COOH

MS

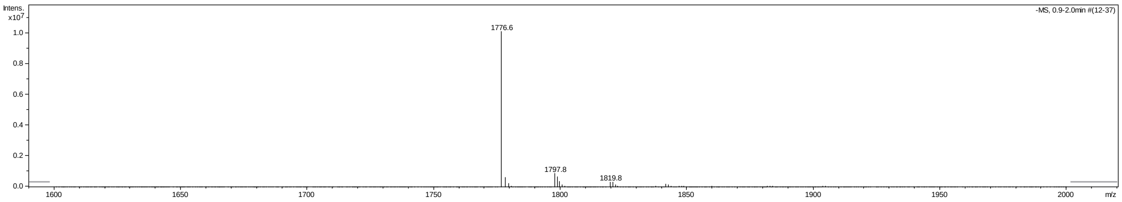

Note S12. SLP4: Ac-VVVTLLLEEEK(Cy5)-COOH

MS

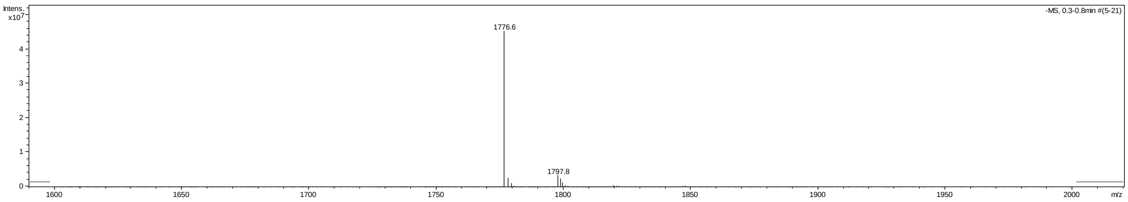

Supplementary References

1 Wassenaar, T. A., Pluhackova, K., Böckmann, R. A., Marrink, S. J. & Tieleman, D. P. Going Backward: A Flexible Geometric Approach to Reverse Transformation from Coarse Grained to Atomistic Models. *Journal of Chemical Theory and Computation* **10**, 676-690, doi:10.1021/ct400617g (2014).

2 Wimley, W. C., Creamer, T. P. & White, S. H. Solvation Energies of Amino Acid Side Chains and Backbone in a Family of Host−Guest Pentapeptides. *Biochemistry* **35**, 5109-5124, doi:10.1021/bi9600153 (1996).
