# Supplementary material for: Control over the fibrillization yield by varying the oligomeric nucleation propensities of self-assembling peptides
Source: Commun Chem. 2020 Nov 11;3:164. doi: 10.1038/s42004-020-00417-7 (PMC9814929; doi:10.1038/s42004-020-00417-7)
Supplement: Supplementary file 2 — Description of Additional Supplementary Files [file 42004_2020_417_MOESM2_ESM.pdf]

Description of Additional Supplementary Files

File Name: Supplementary Data 1
Description: The MD trajectories described in the manuscript
